# Accelerated Synthesis of Graphene Oxide from Graphene

**DOI:** 10.3390/nano11020551

**Published:** 2021-02-22

**Authors:** Mariana C. F. Costa, Valeria S. Marangoni, Pei Rou Ng, Hang T. L. Nguyen, Alexandra Carvalho, A. H. Castro Neto

**Affiliations:** 1 Centre for Advanced 2D Materials, National University of Singapore, Singapore 117456, Singapore; mariana.cfcosta@u.nus.edu (M.C.F.C.); valeriamarangoni@nus.edu.sg (V.S.M.); c2dnpr@nus.edu.sg (P.R.N.); lehang@gmail.com (H.T.L.N.); carvalho@nus.edu.sg (A.C.); 2 Department of Materials Science and Engineering, National University of Singapore, Singapore 117575, Singapore

**Keywords:** graphene oxide, synthesis, graphene, degree of oxidation

## Abstract

Graphene oxide (GO) is an oxygenated functionalized form of graphene that has received considerable attention because of its unique physical and chemical properties that are suitable for a large number of industrial applications. Herein, GO is rapidly obtained directly from the oxidation of graphene using an environmentally friendly modified Hummers method. As the starting material consists of graphene flakes, intercalant agents are not needed and the oxidation reaction is enhanced, leading to orders of magnitude reduction in the reaction time compared to the conventional methods of graphite oxidation. With a superior surface area, the graphene flakes are quickly and more homogeneously oxidized since the flakes are exposed at the same extension to the chemical agents, excluding the necessity of sonication to separate the stacked layers of graphite. This strategy shows an alternative approach to quickly producing GO with different degrees of oxidation that can be potentially used in distinct areas ranging from biomedical to energy storage applications.

## 1. Introduction

The presence of functional groups attached to graphene extends the functionalities of this two-dimensional (2D) material. Graphene oxide (GO) is an important form of functionalized graphene that disperses in water and other polar solvents. Because of the presence of oxygenated groups, such as carboxyl, hydroxyl, and epoxy, GO can be further functionalized with various organic molecules, making it a versatile chemical functionalization platform with an extensive potential for a wide range of applications in some of the greatest challenges of our time, such as water treatment, energy storage, environment, and medicine [1,2].

Beyond the chemical synthesis, the choice of the carbon source also plays an important role in the final quality of GO. Specifically, different types of graphite and synthesis methods can lead to significant modifications in the physical characteristics of GO, such as the lateral size of the flake, the number of layers, and disorder. As a result, the final properties of GO are sensitive to the materials and methods of production, making every structure suitable for specific applications. For example, highly oxidized GO flakes with sub-micrometer size are of great interest for biomedical applications [3], whereas larger GO flakes and a lower degree of oxidation are commonly used in nanocomposites [4].

The Hummers method is the most-used approach for preparing GO and consists of the oxidation of graphite using potassium permanganate (KMnO_4_) and sodium nitrate (NaNO_3_) in a concentrated sulfuric acid (H_2_SO_4_) solution [5]. Despite its popularity, this method presents several drawbacks, including long reaction times for oxidation, up to hundreds of hours, and the formation of toxic gasses such as nitrogen dioxide/dinitrogen tetroxide (NO_2_/N_2_O_4_) due to the presence of NaNO_3_. Additionally, the Hummers method uses not only large amounts of concentrated H_2_SO_4_ and KMnO_4_ to ensure sufficient oxidation of graphite flakes but also huge amounts of water to remove the excessive ions after oxidation. As a result, the process is costly in terms of time, energy, and waste treatment, with several safety and health concerns. Besides, the oxidation is not homogeneous, with the oxidation degree being extremely difficult to control, and some residual graphite is still left unless long sonication periods are used, to produce just a few layers of GO [6].

Recently, numerous strategies have been developed to minimize the issues mentioned above. For example, to reduce the oxidation time, stronger oxidizing agents, such as potassium ferrite (K_2_FeO_4_) [7] or electrochemistry approaches, have been used [8]. Other strategies exclude the usage of NaNO_3_ to avoid the formation of toxic gasses and to facilitate the disposal of wastes due to the absence of Na^+^ and NO_3_^−^ ions. However, the elimination of NaNO_3_ usually demands compensatory strategies to guarantee the efficiency of the Hummers method. Reports including increased amounts of KMnO_4_ and H_2_SO_4_, the introduction of new components, such as phosphoric acid (H_3_PO_4_), to the reaction [9], or reactions with elevated temperatures (as high as 90 °C and above) [5] have been observed.

**Figure 1:**
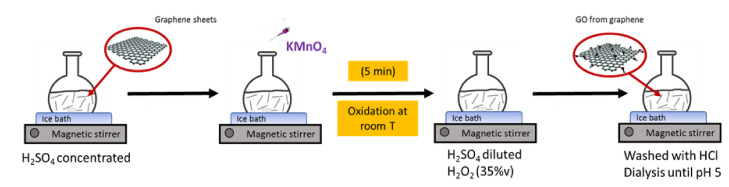
Schematic representation of the rapid synthesis (5 min of oxidation) of graphene-oxide obtained directly from graphene sheets.

The liquid phase exfoliation (LPE) in organic solvents, such as *N*-methyl-2-pyrrolidone, is used to produce monolayer graphene and platelets originally from graphite on large scales [10]. The direct oxidation of graphene flakes obtained by LPE [11] allows us to reduce not only the reaction time, but also the chemically aggressive conditions required for the oxidation of graphite, leading to a much more controllable introduction of oxygenated functional groups on the graphene surface. Over the past few years, the oxidation of graphene flakes has been demonstrated via photo irradiation of graphene in a suspension and via chemical methods [12,13]. Herein, we demonstrate that GO with a controllable degree of oxidation can be rapidly produced by a more environmentally friendly simplified Hummers method. The novelty of this work relies on the use of high-quality graphene sheets instead of graphite as the starting material to produce GO with micrometer lateral dimensions and a high oxidation degree. The higher surface area of unstacked graphene sheets in comparison with pilled layers of graphite favors accelerated oxidation reactions. As NaNO_3_ is not required, this chemical route is considered eco-friendlier and much less harmful to health and the environment compared to the traditional modified Hummers method. Figure 1 compares the starting material used in our method (graphene sheets) with the traditional modified Hummers method (graphite) and shows the synthesis to produce GO directly from graphene. Two main aspects should be highlighted in our approach: the absence of NaNO_3_ and, consequently, the absence of toxic gasses (NO_2_/N_2_O_4_) and the reduced oxidation time.

## 2. Materials and Methods

### 2.1. Synthesis of Graphene Oxide Obtained Directly from Graphene

Graphene flakes were supplied by 2D Materials Pte. Ltd. (2DM) (Singapore). The chemical route to obtain graphene oxide directly from graphene consisted of an environmentally friendly simplified Hummers method in which 0.5 g of graphene flakes were added to 17 mL of concentrated H_2_SO_4_ (Sigma Aldrich, St. Louis, MO, USA), with subsequent cooling up to 2 °C. Next, 2.25 g of KMnO_4_ (Sigma Aldrich, St. Louis, MO, USA) was slowly added to the suspension. The system was kept under stirring at room temperature for different periods of time, in which the graphene flakes were oxidized, followed by cooling to 2 °C and further dilution in water. Lastly, the resulting GO suspensions were cleaned with 3 cycles of washing using HCl (Sigma Aldrich, St. Louis, MO, USA) 10% and dialysis until pH 5.

### 2.2. Commercial Graphene Oxide

GO with different degrees of oxidations were obtained from Sigma Aldrich (St. Louis, MO, USA) and Abalonyx (Oslo, Norway). The materials were used without further purification and are labelled as commercial GO I and II (c-GO I and c-GO II, respectively).

### 2.3. Characterization Techniques

The samples were drop casted on silicon (Si) substrates for XPS and SEM analyses and on silicon substrate with 300 nm silicon dioxide (Si/SiO_2_) for Raman spectroscopy and atomic force microscopy (AFM). For XPS analysis, the measurements were performed in a Kratos AXIX Ultra (Kratos Analytical Ltd., Kyoto, Japan) equipment with a mono-chromatic source Al Kahv = 1486.81 eV. The calibration, Shirley-type background, peak fitting, and quantification were carried out using Casa-XPS software (version 2.1.19) (Casa XPS, Japan). For the SEM analysis, a FESEM VERIOS 460 (FEI Company, Hillsboro, OR, USA) with an accelerating voltage of 2.0 kV and a current of 100 pA was used. For Fourier transform infrared (FTIR) spectroscopy, an ALPHA Platinum-ATR (Bruker Corporation, Billerica, MA, USA), instrument was used. For this, the aqueous suspensions of GO were freeze-dried, and prepared by the KBr disc method, and the spectra were obtained in a in attenuated total reflection (ATR) mode. Atomic force microscopy (AFM) measurements were carried out in a Bruker Dimension Icon Microscope (Bruker Corporation, Billerica, MA, USA) operated in ScanAsyst tapping mode and scan lines of 512 under ambient conditions. Confocal Raman spectroscopy was performed in a WITec Alpha 300R (WITec Wissenschaftliche Instrumente und Technologie GmbH, Germany) with an excitation wavelength of 532 nm and a 100× objective.

### 2.4. Computational Methods

We modelled the main different functional groups present in graphene oxide and calculated their vibrational frequencies from first-principles calculations for comparison with the FTIR absorption bands. First-principles calculations were based on the framework of Density Functional Theory (DFT), as implemented in Quantum ESPRESSO v. 6.5 [14], with the Perdew-Burke-Ernzerhof (PBE) [15,16] exchange and correlation functional. Ultra-soft pseudo-potentials were used for carbon and oxygen [17], while a norm-conserving Troullier–Martins pseudo-potential was used for hydrogen [18]. We employed a plane wave basis set with kinetic energy cutoffs of 40 Ry for the wave functions. The Brillouin zone was sampled using a Γ-centered 6 × 6 × 1 Monkhorst-Pack (MP) grid [19] for all calculations. A supercell periodicity of 20 Angstrom in the direction perpendicular to the monolayer was used to avoid spurious interactions between replicas. Both supercell and flake models were used, and the vibrational modes for different functional groups were obtained by diagonalizing the dynamical matrix for a select number of neighboring atoms.

## 3. Results

**Figure 2:**
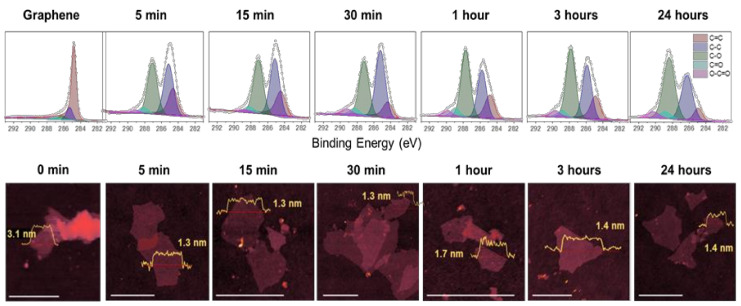
Characterization of graphene and GO obtained directly from graphene flakes. High resolution XPS C1s spectra for graphene and GO after 5, 15, and 30 min, and 1, 3, and 24 h with their respective atomic force microscopy (AFM) images and inset height profiles. Scales bar are 1 µm.

The evolution of the increase in oxygenated groups in the graphene structure as a function of the oxidation time is demonstrated in Figure 2. After the deconvolution of the X-ray photoelectron spectroscopy (XPS) spectra, five main peaks were identified: 284.8 (C=C), 285.7 (C–C), 287.7 (C–O), 288.8 (C=O), and 289.8 eV (O–C=O). After only 5 min of oxidation, we observed a significant relative increase in the oxygen-based groups, indicating that the process of oxidation of graphene sheets is efficient and extremely fast due to the exposure of a higher surface area of graphene. Although not as pronounced, by further increasing the oxidation time, these oxygen-based functional groups continued to increase. However, most of the oxidation process occurred in the first minutes (or even shorter time scale) of reaction.

The thickness and lateral size of GO obtained from atomic force microscopy (AFM) images did not change significantly after 5 min of reaction, indicating that most of the modifications occur very quickly, beyond our limit of observation due to the restrictions of the experimental setup conditions. Looking closer at the spectra and comparing the percentages shown in Table 1, we also noted changes in the relative quantity of each group. For example, the ratio between the O–C=O and C–O groups was greater after 24 h compared to 5 min, indicating that after longer periods of oxidation, the graphene structure is saturated with epoxy groups. Comparing the XPS C1s C–O peak position with first-principles calculations [20], we estimated that the C:O ratio was close to 2:1.

**Figure 3:**
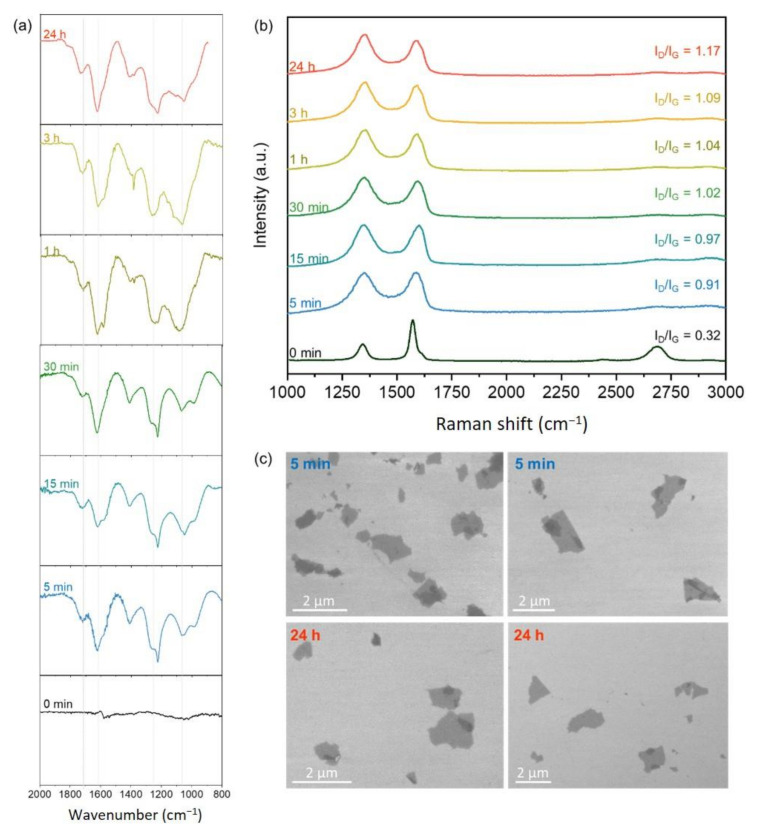
Characterization of graphene and GO obtained directly from the oxidation of graphene. (**a**) FTIR and (**b**) Raman spectra for different periods of oxidation. (**c**) SEM images comparing 5 min and 24 h of oxidation reaction.

Figure 3a shows the evolution of the FTIR spectra for graphene and GO obtained directly from graphene as a function of the oxidation time. For GO, the spectra are typical for this oxygenated functionalized graphene structure, in which the resonances around 1054, 1260, and 1418 cm^−1^ are attributed to the C–OH (alkoxy) stretching, C–O (epoxy) stretching, and O–H (hydroxyl) deformation vibrations, respectively. These are compared with first- principles calculations, which find C–OH bending modes at 1089–1095 cm^−1^, the epoxy symmetric stretch at 1254 cm^−1^, and C–OH hydrogen wag mode at 1509 cm^−1^, which may be the origin of the resonance observed at 1418 cm^−1^. The carboxylic acid group appears at 1733 cm^−1^ with the carbonyl (C=O) stretching calculated to lie at 1691 cm^−1^. The peak at 1620 cm^−1^ is usually attributed to the non-oxidized domains in the graphene structure [21,22], but it can also contain contributions of the adsorbed water molecules [23]. We found from first-principles calculations that the hydroxyl at the flake edge also has two modes in this region, calculated to lie at 1596 and 1615 cm^−1^. These modes are very close in frequency with a graphene vibrational mode and therefore have significant localization in the carbon atoms as well (Table S1) [23]. Specifically, the contribution of the OH from the water molecules occurs at 3200 cm^−1^, and the band at 1620 cm^−1^ increased with the oxidation time, as previously reported [24]. We observed that the increase in oxygen-related bands was pronounced after only 5 min of oxidation. The relative intensity of the groups’ changes is also in agreement with the XPS results.

As expected, after the oxidation, the degree of disorder of the graphene backbone was higher, corroborating the higher relative intensity of the D band and a broader G band in the Raman spectra (Figure 3b). After 5 min of oxidation, we observed a significant difference in the relative intensities, full width at half maximum (FWHM), and I_D_/I_G_ of the D and G GO modes (Table S3). As the oxidation time increased, a slight increase in the I_D_/I_G_ ratio was observed. The 2D band at ~2683 cm^−1^ is related to the number of layers of graphene and their relative orientations [25]. The graphene spectrum is typical for few-layer graphene, and this is expected due to the sample preparation (dispersion in isopropyl alcohol (IPA) and drop-cast on Si/SiO_2_ substrate), which can result in aggregation. After the oxidation, the sharp 2D band at ~2683 cm^−1^ disappears, and some broad features between 2600 and 3000 cm^−1^, typical for GO [26], can be observed. The scanning electron microscopy (SEM) images (Figure 3c) demonstrate that the morphology of the structures did not change significantly from 5 min to 24 h of oxidation reaction. Since graphene has a higher surface area compared to graphite, the use of sonication to complete the exfoliation process is not necessary, and the structure is preserved from the beginning [11] to the end of the synthesis (Figure S1).

By comparing our GO obtained directly from graphene with two commercially available GO obtained from graphite (Figures S2 and S3), we see that their chemical compositions are extremely similar (see Table 1 and Table 2 for details).

However, as expected, GO from graphite presents larger sheets (lateral size) compared to GO from graphene since the graphene sheets used in this study had a small lateral size of 1 µm (Figure 2). Importantly, as graphene layers are already unstacked before oxidation, our method does not require sonication to further separate stacked layers after the introduction of oxygenated functional groups, which prevents extra fractures and defects on the GO structure. Consequently, the original lateral size of graphene flakes is preserved even for highly oxidized structures, as indicated in the size distribution analysis (Figure S3).

## 4. Discussion

We proposed an approach to synthesize graphene oxide with a controllable degree of oxidation. The route consists of an environmentally friendly modified Hummers method, in which the carbon source is graphene, not graphite flakes as in the standard processes. For this reason, intercalant agents, such as NaNO_3_, that are commonly used to expand the graphite structure are not required, and toxic gasses, such as NO_2_/N_2_O_4_, are not generated, diminishing several safety and health concerns. Consequently, the reaction time is reduced by the orders of magnitude when compared with conventional methods. As graphene has a comparatively larger surface area per volume, the oxygenated groups are rapidly and homogeneously distributed over the graphene lattice, eliminating steps of sonication to further separate the layers.

## 5. Conclusions

Our study opens up new avenues to explore environmentally friendly production routes as well as novel mild oxidation approaches to expand industrial applications of GO, such as chemical sensors [27], solar cells [28], nanocomposite materials [29,30], energy storage [31], and biomedical applications [32].

## Figures and Tables

**Table 1:** Binding energy of the deconvoluted XPS C1s peaks and their relative percentage areas (in parentheses) for the spectra shown in Figure 2 and Figure S2.

Material	C=C (sp^2^)	C–C (sp^3^)	C–O	C=O	O–C=O	π → π *
Graphene	284.76 (81.8%)	285.30 (10.5%)	286.24 (2.8%)	286.99 (1.4%)	287.77 (0.7%)	290.90 (2.8%)
GO 5 min	284.6 (26.7%)	285.15 (33.6%)	287.08 (33.5%)	288.17 (4.0%)	289.10 (2.1%)	
GO 15 min	284.41 (26.3%)	285.15 (35.7%)	287.11 (33.3%)	288.18 (3.4%)	288.86 (1.3%)	
GO 30 min	284.47 (23.8%)	285.25 (36.8%)	287.15 (31.6%)	288.07 (4.5%)	289.17 (3.3%)	
GO 1 h	284.82 (22.1%)	285.77 (29.4%)	287.71 (40.4%)	288.93 (4.9%)	289.86 (3.2%)	
GO 3h	284.89 (21.7%)	285.89 (31.5%)	287.83 (39.9%)	288.85 (3.6%)	289.68 (3.3%)	
GO 24 h	284.94 (9.4%)	286.26 (34.3%)	288.39 (46.6%)	288.79 (5.3%)	290.26 (4.4%)	
c-GO I	284.32 (23.6%)	285.03 (33.3%)	286.94 (28.5%)	287.41 (10.9%)	288.34 (3.7%)	
c-GO II	284.81 (19.0%)	285.67 (26.0%)	287.55 (45.1%)	288.59 (6.7%)	289.49 (3.2%)	

**Table 2:** Peak position, FWHM, and I_D_/I_G_ ratio of the D and G bands of GO for the spectra shown in Figure 3b and Figure S3a.

Material	D Band		G Band		I_D_/I_G_
	Center	FWHM	Center	FWHM	
Graphene	1343.3 ± 0.4	54.8 ± 1.0	1570.5 ± 0.2	26.6 ± 0.3	0.32
GO 5 min	1355.5 ± 1.0	166.0 ± 3.0	1578.3 ± 1.0	101.0 ± 2.0	0.91
GO 15 min	1345.5 ± 1.0	145.0 ± 3.0	1587.0 ± 1.0	101.0 ± 2.0	0.97
GO 30 min	1352.5 ± 1.0	175.0 ± 3.0	1584.9 ± 0.9	102.0 ± 2.0	1.02
GO 1 h	1354.1 ± 1.0	167.2 ± 2.0	1582.5 ± 0.8	98.8 ± 2.0	1.04
GO 3 h	1354.9 ± 1.0	164.0 ± 2.0	1582.8 ± 0.9	98.5 ± 2.0	1.09
GO 24 h	1352.4 ± 1.0	162.0 ±2.0	1581.0 ± 0.8	100.8 ± 2.0	1.17
c-GO I	1368.5 ± 1.0	171.9 ± 3.0	1585.0 ± 0.7	105.7 ± 1.0	1.00
c-GO II	1365.5 ± 3.0	154.5 ± 8.0	1584.5 ± 2.0	108.9 ± 1.0	0.89

## Supplementary Materials

The following are available online at https://www.mdpi.com/2079-4991/11/2/551/s1: Figure S1: Lateral size distributions for graphene flakes after 5 min and 24 h of oxidation; Figure S2: Characterization of commercial GO obtained. High resolution C1s XPS spectra and their respective AFM images. Scales bar are 2 m. Figure S3: Characterization of commercial GO. (a) Raman, and (b) FTIR spectra; Table S1: Calculated local vibrational modes (LVMs) of oxygen and hydrogen functional groups in graphene. Only vibrational modes with frequencies above 600 cm^−1^ and with localization (loc.) on O and H of 2% or larger are shown. Basal plane functional groups were modelled using a supercell model and edge functional groups were modelled using a graphene flake model.
