# Supplementary material for: Accelerated Synthesis of Graphene Oxide from Graphene
Source: Nanomaterials (Basel). 2021 Feb 22;11(2):551. doi: 10.3390/nano11020551 (PMC7926456; doi:10.3390/nano11020551)
Supplement: Supplementary file 1 [file nanomaterials-11-00551-s001.pdf]

nanomaterials

Supplementary Materials: Accelerated Synthesis of Graphene Oxide from Graphene

Mariana C. F. Costa [1,2,†], Valeria S. Marangoni [1,†], Pei Rou Ng [1], Hang T. L. Nguyen [1], Alexandra Carvalho [1] and A. H. Castro Neto [1,2,*]

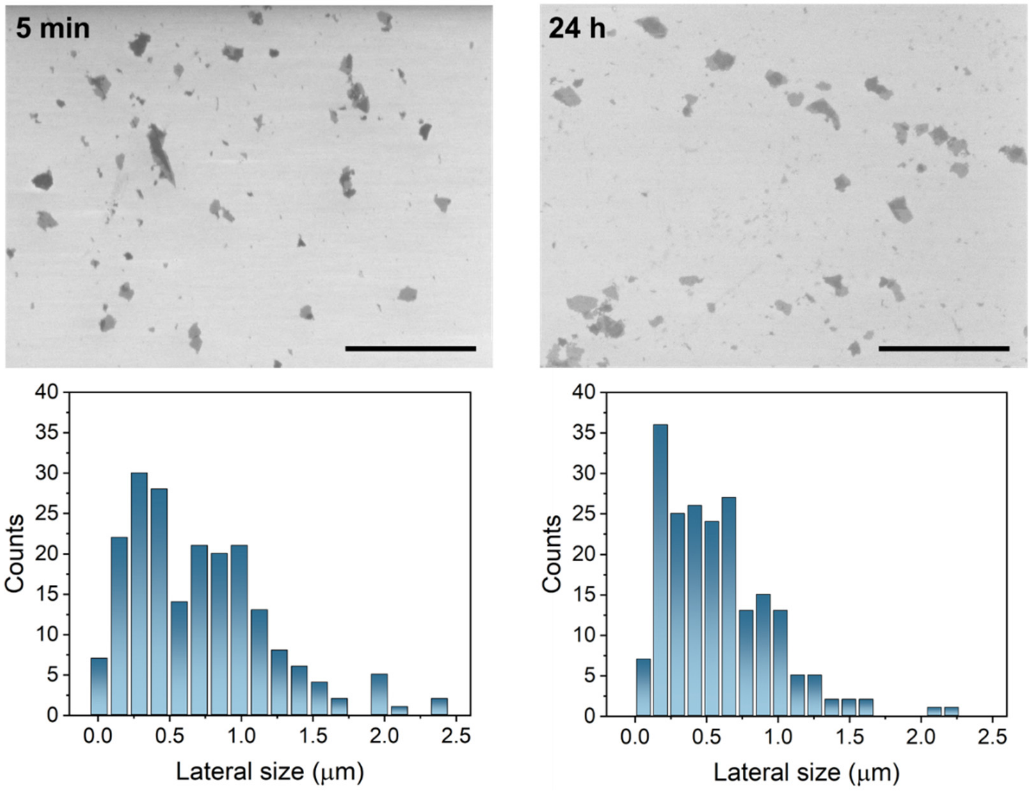

Figure S1. Lateral size distributions for GO flakes after 5 min and 24 h of oxidation.

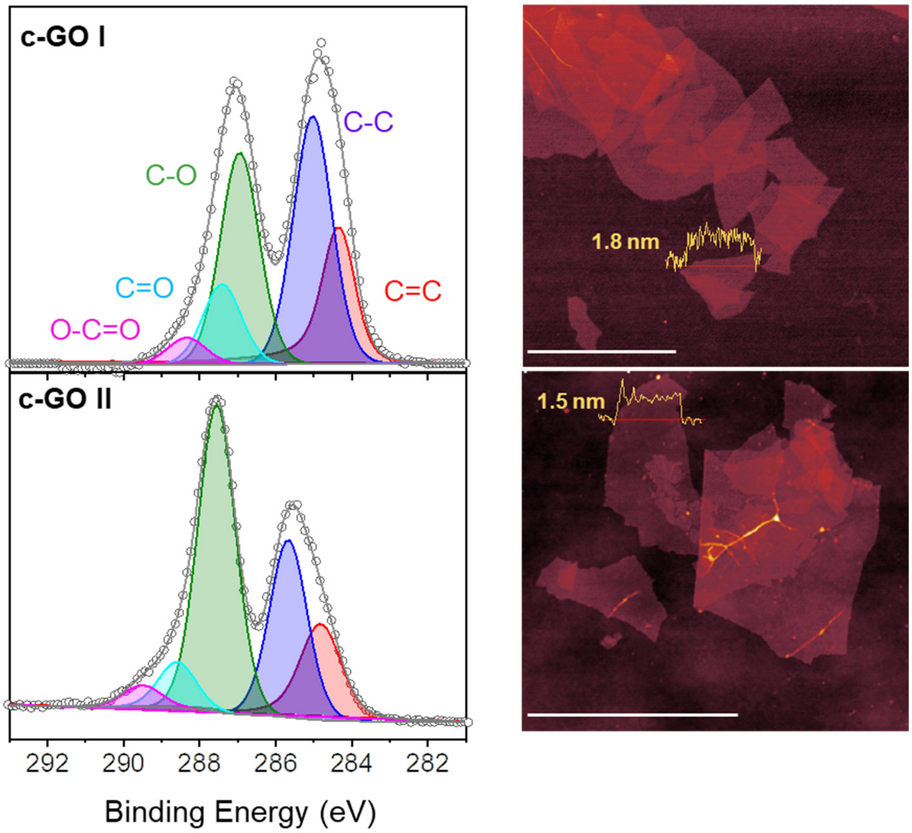

Figure S2. Characterization of commercial GO obtained. High resolution C1s XPS spectra and their respective AFM images. Scales bar are 2 µm.

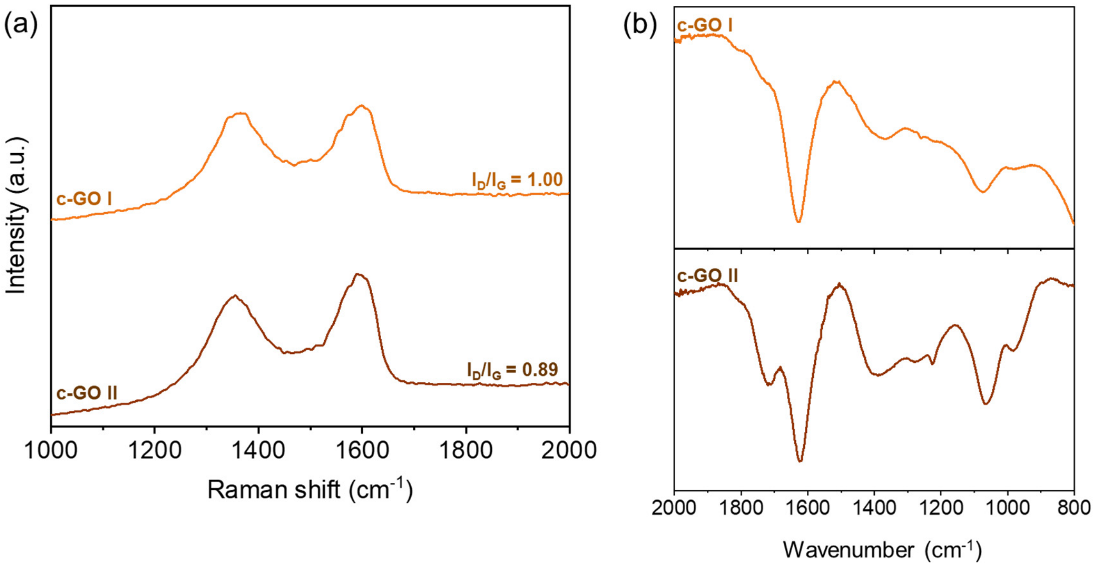

Figure S3. Characterization of commercial GO. (**a**) Raman, and (**b**) FTIR spectra.

Table S1. Calculated local vibrational modes (LVMs) of oxygen and hydrogen functional groups in graphene. Only vibrational modes with frequencies above 600 cm^{-1} and with localization (loc.) on O and H of 2% or larger are shown. Basal plane functional groups were modelled using a supercell model and edge functional groups were modelled using a graphene flake model.

**Functional Group**	**Symbol**	**Model**	**O/H-LVMs (cm^{-1})**	**Loc. (%)**
Epoxy	>O	Supercell	1254	2.8
			892	2.3
			801	4.1
			717	3.9
			699	2.0
			696	2.0
			614	4.7
Hydroxyl (surface)	–OH	Supercell	3632	10.0
			1509	2.0
			771–1319	>.0
Hydroxyl (edge)	–OH	flake	3575	10.0
			1615	2.1
			1596	2.1
			1382–1488	>2.0
			1109	7.5
			1039	2.4
			814	2.8
			775	2.5
			651	2.1
Carbonyl	–COOH		3508	9.9
			1692	5.4
			1230–1335	>2.0
			1149	6.0
			778–997	>2.0
			671–732	>2.0
